# Isolation of Alpha-Toxin-Deficient Clostridium perfringens Type F from Sewage Influents and Effluents

**DOI:** 10.1128/spectrum.00214-21

**Published:** 2021-07-14

**Authors:** Keita Yanagimoto, Eiji Haramoto

**Affiliations:** a Department of Microbiology, Yamanashi Institute of Public Health and Environment, Kofu, Yamanashi, Japan; b Interdisciplinary Center for River Basin Environment, University of Yamanashi, Kofu, Yamanashi, Japan; The Pennsylvania State University

**Keywords:** alpha-toxin, phospholipase C negative, lecithinase negative, wastewater treatment plant, multilocus sequence typing

## Abstract

Clostridium perfringens is classified into types A to G, and all types produce alpha-toxins; however, C. perfringens type F that is negative for phospholipase C (PLC) activity of alpha-toxin has been isolated from the environment and cases of humans afflicted by food poisoning. This study aimed to elucidate the distribution of PLC-negative C. perfringens type F in sewage influents and effluents. Influents and effluents of two wastewater treatment plants were collected monthly between July 2016 and January 2020 and between August 2018 and January 2020, respectively. Isolation rates of PLC-negative C. perfringens type F from sewage influents and effluents were 38% (33/86) and 22% (8/36), and the numbers of isolates were 43 and 13, respectively. The locus of the enterotoxin gene of all isolates was determined to be in a plasmid with an IS*1151* sequence, and multilocus sequence typing revealed that all 17 representative isolates were assigned as sequence type 186. Sequencing of the *plc* gene of these representative isolates showed that nonsense mutation (p.W98*) causing alpha-toxin deficiency should be responsible for a loss of PLC enzymatic activity. These results suggest that alpha toxin-deficient C. perfringens type F is distributed in living and water environments since sewage influents contain community wastewater, and effluents contaminate the environment. Detection of C. perfringens type F, independent of PLC activity, should be carried out on human and environmental samples.

**IMPORTANCE** Understanding the diversity of biochemical characteristics that may affect the identification of bacteria is essential. C. perfringens is a ubiquitous bacterium found in the environment, humans, and animals and is responsible for infectious disease in the intestine. Although the alpha-toxin of C. perfringens may be used for its detection, variants of the alpha-toxin lacking its activity have been isolated from soil and humans experiencing symptoms of diarrhea. It is valuable to disclose the prevalence of the alpha-toxin variant in the sewage of wastewater treatment plants, as it may reflect the hygienic condition of the community, as it would be a pollution source for the environment. This study shows the persistent existence and genetic characteristics of the alpha-toxin variant in sewage and reveals a lacking mechanism of the alpha-toxin activity and proposes the detection method of C. perfringens, independent of the alpha-toxin activity.

## INTRODUCTION

Clostridium perfringens is an anaerobic, spore-forming bacterium that is classified as type A to G based on toxin production, including alpha, beta, epsilon, iota, the C. perfringens enterotoxin (CPE), and NetB toxin production (1). C. perfringens type F produces an alpha-toxin and CPE and is responsible for foodborne and nonfoodborne infection (2, 3). Although all types of C. perfringens produce an alpha-toxin which exhibits phospholipase C (PLC) and sphingomyelinase activity (4), C. perfringens that produces an alpha-toxin negative for PLC activity and is a *plc*-gene variant was isolated from Antarctic soil (5). In addition, previous studies confirmed isolations of PLC-negative C. perfringens type F from food poisoning patients (6, 7). However, these isolates are not typical among human isolates because the detection of PLC activity with egg yolk agar may be used to detect C. perfringens (5, 6, 8); thus, little is known about the distribution of PLC-negative C. perfringens type F among humans and other sources, such as the environment.

In a previous study, Salmonella spp. isolated from sewage influents of wastewater treatment plants (WWTPs) were more variable than those isolated from humans (9). Additionally, genetically variable isolates of C. perfringens type F were recovered from sewage influents and effluents of WWTPs (10, 11). Therefore, sewage may contain variable C. perfringens type F, such as PLC-negative strains. Furthermore, the distribution of PLC-negative strains may be estimated based on sewage samples, which would contain community wastewater and contaminate the water environment (11).

The aim of this study was to reveal the isolation rate, genetic characteristics, and *plc* sequence of PLC-negative C. perfringens type F isolates from sewage influents and effluents of WWTPs to reveal the distribution of these C. perfringens strains in the water environment and to detect the PLC-negative mechanism.

## RESULTS

### Isolation of PLC-negative C. perfringens type F.

The isolation rates of PLC-negative C. perfringens type F from sewage influents and effluents of two WWTPs (WWTP-A and WWTP-B) in Yamanashi, Japan, were 38% (33/86) and 22% (8/36), and the number of isolates from these was 43 and 13, respectively (Table S1 in the supplemental material). All isolates except for two isolates from two sewage influents of WWTP-B were positive for the C. perfringens beta2 toxin gene (*cpb2*). There was no significant difference between WWTP-A and WWTP-B in the isolation rate and number of isolates (*P* > 0.05), and no seasonal trend was observed.

### Characterization of the isolates.

Characterization of the isolates was conducted by *cpe* genotyping to determine the locus of *cpe* and multilocus sequence typing (MLST). The locus of *cpe* of all isolates was discovered in a plasmid with an IS*1151* sequence downstream of *cpe*. MLST demonstrated that all representative 17 isolates were assigned as sequence type (ST) 186 (Table 1).

**TABLE 1:** Characteristics of PLC-negative C. perfringens type F as assessed by MLST

Strain	Location of *cpe*	*cpb2* ^*a*^	Source	WWTP	Mo and yr of isolation	ST
17-97	Plasmid with an *IS1151* sequence	+	Influent	A	April 2017	186
17-113	Plasmid with an *IS1151* sequence	+	Influent	B	April 2017	186
18-67	Plasmid with an *IS1151* sequence	+	Influent	A	December 2017	186
18-104	Plasmid with an *IS1151* sequence	+	Influent	B	January 2018	186
18-108	Plasmid with an *IS1151* sequence	−	Influent	B	February 2018	186
18-266	Plasmid with an *IS1151* sequence	+	Influent	A	August 2018	186
18-331	Plasmid with an *IS1151* sequence	+	Influent	A	October 2018	186
19-37	Plasmid with an *IS1151* sequence	+	Influent	A	February 2019	186
19-41	Plasmid with an *IS1151* sequence	−	Influent	B	February 2019	186
19-98	Plasmid with an *IS1151* sequence	+	Influent	B	April 2019	186
19-198	Plasmid with an *IS1151* sequence	+	Influent	A	November 2019	186
19-195	Plasmid with an *IS1151* sequence	+	Influent	B	November 2019	186
18-280	Plasmid with an *IS1151* sequence	+	Effluent	A	October 2018	186
18-283	Plasmid with an *IS1151* sequence	+	Effluent	B	October 2018	186
18-348	Plasmid with an *IS1151* sequence	+	Effluent	A	January 2019	186
19-61	Plasmid with an *IS1151* sequence	+	Effluent	B	April 2019	186
19-203	Plasmid with an *IS1151* sequence	+	Effluent	B	December 2019	186

a +, positive; −, negative.

### Analysis of the *plc* sequence.

Analysis of the *plc* sequence was performed to determine the PLC-negative mechanism and alpha-toxin sequence type. The sequencing of *plc* revealed that all isolates were identical to each other and showed a nucleotide substitution at nucleotide position 293 (c.293G>A) compared with the *plc* sequence of CP228 (12) (Fig. S1). This nucleotide substitution resulted in a nonsense mutation (p.W98*) in the deduced amino acid sequence, which was determined to be a single amino acid substitution of the alpha-toxin sequence type Ve and was designated alpha-toxin sequence type N (nonsense mutation) (Table 2 and Fig. S2).

**TABLE 2:** Alpha-toxin sequence types and their amino acid positions that were different from those of strain 13

Alpha-toxin sequence type^*a*^	Amino acid at position:^*b*^												
	13	15	22	47	54	71	98	149	195	202	205	365	373
Strain 13	T	A	A	V	L	E	W	L	A	D	A	A	I
Type I	A												
Type II	A												V
Type III		V	V		M	D		I					V
Type IV	A				M								V
Type V	A									A	T		
Type Ve	A									A	T	V	V
Type VI	A								V	N			V
Type VII	A		S	I					V				V
Type VIII													V
Type IX				I						A			V
Type X				I									L
Type N^*c*^	A						*^e^			(A)^*d*^	(T)^*d*^	(V)^*d*^	(V)^*d*^

*^a^*Alpha-toxin sequence type strain 13 and types I to V, type Ve, and types VI to X were defined by Sheedy et al. (19), Matsuda et al. (12), and Ablidgaard et al. (20), respectively.

*^b^*Amino acid positions different from those of strain 13.

*^c^*Type N (nonsense mutation) as defined in the present study.

*^d^*Amino acid in parentheses indicates the deduced amino acid if there was not a stop codon at position 98.

*^e^*Asterisk shows a stop codon.

## DISCUSSION

In the present study, we revealed the presence and genetic characteristics of PLC-negative C. perfringens type F isolates from sewage influents and effluents of WWTPs and elucidated their PLC-negative mechanism. Although PLC-negative C. perfringens has rarely been reported since PLC activity is an important criterion for the identification of C. perfringens (5, 6, 8), the present study showed that PLC-negative C. perfringens type F was isolated from 38% and 22% of sewage influents and effluents, respectively. Our previous study revealed that the isolation rates of PLC-positive C. perfringens type F from sewage influents and effluents were 80% (69/86) and 56% (20/36), respectively (11), which was higher than that of PLC-negative C. perfringens type F observed in the present study. These results suggest that even though PLC-negative isolates occur less among C. perfringens type F strains, the presence of PLC-negative isolates in water environments such as sewage should be considered.

The *plc* sequencing of all isolates showed a nucleotide substitution (c.293G>A) compared with the *plc* sequence of CP228 (12), and this mutation caused a nonsense mutation (p.W98*) in the deduced amino acid sequence. The alpha-toxin consists of two domains, an N-terminal domain (amino acids 1 to 246), which is responsible for enzymatic activity, and a C-terminal domain (amino acids 256 to 370), which is responsible for binding to the membrane and required for hemolytic activity (4). Alpha-toxin deficiency in the present study was estimated to include part of the N-terminal domain (1 to 97) and no C-terminal domain. These results demonstrate that the PLC-negative mechanism of the isolates potentially occurs due to an alpha-toxin deficiency that is caused by p.W98*, which leads to the loss of enzymatic activity of PLC.

In the present study, alpha-toxin-deficient isolates possess an alpha-toxin designated alpha-toxin sequence type N, which has a single amino acid substitution of alpha-toxin sequence type Ve, which is the most prevalent alpha-toxin sequence type in humans in Hokkaido, Japan (12). Although there is a possibility that the alpha-toxin sequence type N is most prevalent among alpha-toxin-deficient isolates in Japan due to these results and the isolation rate of isolates in the present study, more investigations about the pathogen are necessary to discuss the situation.

Although representative isolates were examined by MLST, the results of the *cpe* genotyping assay and MLST indicated that the locus of *cpe* and the ST of the isolates in the present study were identical (a plasmid with IS*1151* sequence and ST186, respectively), which indicates that strains with common characteristics are widely distributed in water environments. When this is not the case, these strains show higher resistance than other alpha-toxin-deficient C. perfringens type F against environmental stress in sewage pipes and WWTPs, even though it is not identified whether a nonsense mutation of the isolates is related to this resistance. We believe that both hypotheses occur simultaneously since it is unlikely that these strains with the same sequence type (ST) flowed into two sewage pipes independently without wide distribution and were not detected from sewage effluents, which were processed with sewage treatments, without higher stress resistance.

When alpha-toxin-deficient C. perfringens type F isolates from sewage are derived from the community, it is likely that a substantial amount of alpha-toxin-deficient C. perfringens type F blends into living and water environments. This is supported by the fact that C. perfringens type F isolates from sewage influents and effluents were genetically related to those from humans and retailed bivalves (11), which accumulate C. perfringens in the water environment (13). In addition to this, the locus of *cpe* of all isolates in the present study was a plasmid with an IS*1151* sequence, and Kiu et al. reported that strains with plasmidal *cpe*, including a plasmid with an IS*1151* sequence, were the predominant cause of food poisoning cases by C. perfringens (3). Thus, it should be noted that the detection method of C. perfringens type F regardless of PLC activity could be necessary for samples collected from not only the environment but also humans with gastroenteritis because PLC-negative C. perfringens type F was isolated from food poisoning patients (6, 7), and the isolates in the present study may possess a potential for association with gastroenteritis. Future investigations that disclose the distribution of alpha-toxin-deficient C. perfringens type F in other regions would indicate the nature of this pathogen.

A multiplex PCR for the detection of toxin genes revealed that all isolates in our study were positive for the *plc* gene; however, PLC production of all isolates on CW agar plates was negative. This contradiction can be explained by the single-nucleotide substitution of *plc*, which caused the alpha-toxin deficiency and keeps its sensitivity to primers, which enables *plc* detection. This result reminds us that a *plc*-positive strain does not mean that it is a PLC-producing strain.

The limitation of our study is that the genetic comparison of the isolates was conducted by only MLST. Whole-genome sequencing of the isolates would be required for the strict genetic comparison. Additionally, no isolate from human was analyzed in the present study. Further analyses will be essential to show the actual situation in humans.

In conclusion, alpha-toxin-deficient C. perfringens type F was isolated from 38% and 22% of sewage influents and effluents, respectively, and the results of the MLST and *cpe* genotyping assay of all tested isolates were identical. Additionally, *plc* sequencing revealed a nonsense mutation that was estimated to be responsible for the alpha-toxin deficiency. These results suggest that alpha-toxin-deficient C. perfringens type F is distributed in living and water environments, and the detection of C. perfringens type F with or without PLC activity should be conducted on human and environmental samples.

## MATERIALS AND METHODS

### Sample collection.

Sewage influents and effluents of WWTP-A and WWTP-B, which serve a population of ∼350,000, and the total loads of ∼180,000 m^3^/day of wastewater in Yamanashi, Japan, were collected monthly between July 2016 and January 2020 and between August 2018 and January 2020, respectively. The collected samples were concentrated by centrifugation, as described previously (11).

### Isolation methods for PLC-negative C. perfringens type F.

Concentrated samples were cultured in enrichment broth, as described previously (11). Colonies without PLC production were observed on a CW agar plate (Nissui Pharmaceutical, Tokyo, Japan) that contained 50% egg yolk-enriched saline (Kyokuto, Tokyo, Japan) and were isolated and suspended in sterilized distilled water. Then, DNA was extracted by heating at 100°C for 10 min, and PCR with species-specific primers based on the 16S rRNA gene of C. perfringens was conducted for identification of the isolates as described by Kikuchi et al. (14). Detection of toxin genes, including C. perfringens alpha, beta, epsilon, iota, and *cpb2*, and *cpe*, was performed as described by van Asten et al. (15) (Table 3).

**TABLE 3:** Primers used in this study

Primer	Sequence (5′–3′)	Application	Reference
CIPER-F	AGATGGCATCATCATTCAAC	Identification of C. perfringens	14
CIPER-R	GCAAGGGATGTCAAGTGT		
CPAlphaF	GCTAATGTTACTGCCGTTGA	Toxinotyping	15
CPAlphaR	CCTCTGATACATCGTGTAAG		
CPBetaF3	GCGAATATGCTGAATCATCTA		
CPBetaR3	GCAGGAACATTAGTATATCTTC		
CPBeta2totalF2	AAATATGATCCTAACCAAMAA		
CPBeta2totalR	CCAAATACTYTAATYGATGC		
CPEpsilonF	TGGGAACTTCGATACAAGCA		
CPEpsilonR2	AACTGCACTATAATTTCCTTTTCC		
CPIotaF2	AATGGTCCTTTAAATAATCC		
CpIotaR	TTAGCAAATGCACTCATATT		
CPEnteroF	TTCAGTTGGATTTACTTCTG		
CPEnteroR	TGTCCAGTAGCTGTAATTGT		
cpe4F	TTAGAACAGTCCTTAGGTGATGGAG	*cpe* genotyping	16
IS1470R1.3	CTTCTTGATTACAAGACTCCAGAAGAG		
IS1470-likeR1.6	CTTTGTGTACACAGCTTCGCCAATGTC		
IS1151R0.8	ATCAAAATATGTTCTTAAAGTACGTTC		
3F	GATAAAGGAGATGGTTGGATATTAGG		
4R	GAGTCCAAGGGTATGAGTTAGAAG		
gyrB-F	ATTGTTGATAACAGTATTGATGAAGC	MLST	17
gyrB-R	ATTTCCTAATTTAGTTTTAGTTTGCC		
sigK-F	CAATACTTATTAGAATTAGTTGGTAG		
sigK-R	CTAGATACATATGATCTTGATATACC		
sod-F	CAAAAAAAGTCCATTAATGTATCCAG		
sod-R	TTATCTATTGTTATAATATTCTTCAC		
groEL-F	TACAAGATTTATTACCATTACTTGAG		
groEL-R	CATTTCTTTTTCTGGAATATCTGC		
pgk-F	GACTTTAACGTTCCATTAAAAGATGG		
pgk-R	CTAATCCCATGAATCCTTCAGCGATG		
nadA-F	ATTAGCACATTATTATCAAATTCCTG		
nadA-R	TTATATGCCTTTAATCTTAAATCCTC		
colA-F	ATTAGAAAGTTTATGTACAATAGGTG		
colA-R2	AAGACATTCTATTATTTCTATCGTAAGC		
plc-F	AGGAACTCATGCTATGATTGTAACTC		
plc-R	GGATCATTACCCTCTGATACATCGTG		
ss2	CTTGAAAAAAATTAACGG	*plc* sequencing	19
cpaR	TCTGATACATCGTGTAAG		
cpaF	GCTAATGTTACTGCCGTTGACC		
ss3	TGTAAATACCACCAAAACC		

### Characterization of the isolates.

All isolates were characterized by *cpe* genotyping assay (16) to determine the locus of *cpe*, and 17 representative isolates considering month and year of isolation and source were analyzed by MLST, as described by Deguchi et al. (17) (Table 3). ST of the isolates was determined according to Xiao’s scheme (18) by submitting the sequence data to PubMLST (https://pubmlst.org/organisms/clostridium-perfringens).

### Analysis of *plc* sequence.

To determine the PLC-negative mechanism and the alpha-toxin sequence type, *plc* sequencing of 17 representative isolates was performed. Briefly, DNA was extracted by heating at 100°C for 10 min, and PCR was conducted using Thermal Cycler Dice Touch TP350 (TaKaRa Bio, Kusatsu, Japan). The amplification of *plc* was performed with primer pairs reported by Sheedy et al. (19) (Table 3). Each 25-μl reaction mixture contained 2.5 μl of 10× *Ex Taq* buffer, 2 μl of deoxynucleoside triphosphate (dNTP) mixture, 0.125 μl of TaKaRa *Ex Taq* (TaKaRa Bio), 2 μl each of 2.5-pmol/μl primer, and 2.5 μl of template DNA. PCR products were sequenced with BigDye Terminator v3.1 cycle sequencing kit (Applied Biosystems, Foster City, CA) according to the manufacturer’s instructions. The deduced amino acid sequence of alpha-toxin was obtained using a genetic information processing software (GENETYX ver. 13, Genetyx, Tokyo, Japan), and alpha-toxin sequence type of each isolate was assigned according to previous studies (12, 19, 20).

### Statistical analysis.

The differences between WWTP-A and WWTP-B in the isolation rate and number of isolates were compared using the chi-square test, and a *P* value of <0.05 was considered statistically significant.

### Data availability.

The sequence data obtained in this study were deposited in the DNA Data Bank of Japan and GenBank under accession numbers LC603848 to LC604000.
