# Supplementary material for: Isolation of Alpha-Toxin-Deficient Clostridium perfringens Type F from Sewage Influents and Effluents
Source: Microbiol Spectr. 2021 Jul 14;9(1):10.1128/spectrum.00214-21. doi: 10.1128/spectrum.00214-21 (PMC8552768; doi:10.1128/spectrum.00214-21)
Supplement: SUPPLEMENTAL FILE 1 — Supplemental material. Download SPECTRUM00214-21_Supp_1_seq2.pdf, PDF file, 0.2 MB [file spectrum00214-21_supp_1_seq2.pdf]

```
Strain 13 ATGAAAAGAAAGATTTGTAAGGCGCTTATTTGTGCTACGCTAGCAACTAGCCTATGGGCTGGGGCATCAACTAAAGTCTACGCTTGGGATGGAAAGATTG   100
Type Ve   ATGAAAAGAAAGATTTGTAAGGCGCTTATTTGTGCCGCGCTAGCAACTAGCCTATGGGCTGGGGCATCAACTAAAGTCTACGCTTGGGATGGAAAGATTG   100
Type N    ATGAAAAGAAAGATTTGTAAGGCGCTTATTTGTGCCGCGCTAGCAACTAGCCTATGGGCTGGGGCATCAACTAAAGTCTACGCTTGGGATGGAAAGATTG   100

Strain 13 ATGGAACAGGAACTCATGCTATGATTGTAACTCAAGGGGTTTCAATCTTAGAAAATGATCTGTCCAAAAATGAACCAGAAAGTGTAAGAAAAAACTTAGA   200
Type Ve   ATGGAACAGGAACTCATGCTATGATTGTAACTCAAGGGGTTTCAATCTTAGAAAATGATCTGTCCAAAAATGAACCAGAAAGTGTAAGAAAAAACTTAGA   200
Type N    ATGGAACAGGAACTCATGCTATGATTGTAACTCAAGGGGTTTCAATCTTAGAAAATGATCTGTCCAAAAATGAACCAGAAAGTGTAAGAAAAAACTTAGA   200

Strain 13 GATTTTAAAAGAGAACATGCATGAGCTTCAATTAGGTTCTACTTATCCAGATTATGATAAGAATGCATATGATCTATATCAAGATCATTTCTGGGATCCT   300
Type Ve   GATTTTAAAAGAGAACATGCATGAGCTTCAATTAGGTTCTACTTATCCAGATTATGATAAGAATGCATATGATCTATATCAAGATCATTTCTGGGATCCT   300
Type N    GATTTTAAAAGAGAACATGCATGAGCTTCAATTAGGTTCTACTTATCCAGATTATGATAAGAATGCATATGATCTATATCAAGATCATTTCTAGGATCCT   300

Strain 13 GATACAGATAATAATTTCTCAAAGGATAATAGTTGGTATTTAGCTTATTCTATACCTGACACAGGGGAATCACAAATAAGAAAATTTTCAGCATTAGCTA   400
Type Ve   GATACAGATAATAATTTCTCAAAGGATAATAGTTGGTATTTAGCTTATTCTATACCTGACACAGGGGAATCACAAATAAGAAAATTTTCAGCATTAGCTA   400
Type N    GATACAGATAATAATTTCTCAAAGGATAATAGTTGGTATTTAGCTTATTCTATACCTGACACAGGGGAATCACAAATAAGAAAATTTTCAGCATTAGCTA   400

Strain 13 GATATGAATGGCAAAGAGGAAACTATAAACAAGCTACATTCTATCTTGGAGAGGCTATGCACTATTTTGGAGATATAGATACTCCATATCATCCTGCTAA   500
Type Ve   GATATGAATGGCAAAGAGGAAACTATAAACAAGCTACATTCTATCTTGGAGAGGCTATGCACTATTTTGGAGATATAGATACTCCATATCATCCTGCTAA   500
Type N    GATATGAATGGCAAAGAGGAAACTATAAACAAGCTACATTCTATCTTGGAGAGGCTATGCACTATTTTGGAGATATAGATACTCCATATCATCCTGCTAA   500

Strain 13 TGTTACTGCCGTTGATAGCGCAGGACATGTTAAGTTTGAAACTTTTGCAGAGGAAAGAAAAGAACAGTATAAAATAAACACAGCAGGTTGCAAAACTAAT   600
Type Ve   TGTTACTGCCGTTGATAGCGCAGGACATGTTAAGTTTGAGACTTTTGCAGAGGAAAGAAAAGAACAGTATAAAATAAACACAGCAGGTTGCAAAACTAAT   600
Type N    TGTTACTGCCGTTGATAGCGCAGGACATGTTAAGTTTGAGACTTTTGCAGAGGAAAGAAAAGAACAGTATAAAATAAACACAGCAGGTTGCAAAACTAAT   600

Strain 13 GAGGATTTTTATGCTGATATCTTAAAAAACAAGGATTTTAATGCATGGTCAAAAGAATATGCAAGAGGTTTTGCTAAAACAGGAAAATCAATATACTATA   700
Type Ve   GAGGCTTTTTATACTGATATCTTAAAAAACAAAGATTTTAATGCATGGTCAAAAGAATATGCAAGAGGTTTTGCTAAAACAGGAAAATCAATATACTATA   700
Type N    GAGGCTTTTTATACTGATATCTTAAAAAACAAAGATTTTAATGCATGGTCAAAAGAATATGCAAGAGGTTTTGCTAAAACAGGAAAATCAATATACTATA   700

Strain 13 GTCATGCTAGCATGAGTCATAGTTGGGATGATTGGGATTATGCAGCAAAGGTAACTTTAGCTAACTCTCAAAAAGGAACAGCAGGATATATTTATAGATT   800
Type Ve   GTCATGCTAGCATGAGTCATAGTTGGGATGATTGGGATTATGCAGCAAAGGTAACTTTAGCTAACTCTCAAAAAGGAACAGCAGGATATATTTATAGATT   800
Type N    GTCATGCTAGCATGAGTCATAGTTGGGATGATTGGGATTATGCAGCAAAGGTAACTTTAGCTAACTCTCAAAAAGGAACAGCAGGATATATTTATAGATT   800

Strain 13 CTTACACGATGTATCAGAGGGTAATGATCCATCAGTTGGAAAGAATGTAAAAGAACTAGTAGCTTACATATCAACTAGTGGTGAAAAAGATGCTGGAACA   900
Type Ve   CTTACACGATGTATCAGAGGGTAATGATCCATCAGTTGGAAAGAATGTAAAAGAACTAGTAGCTTACATATCAACTAGTGGTGAAAAAGATGCTGGAACA   900
Type N    CTTACACGATGTATCAGAGGGTAATGATCCATCAGTTGGAAAGAATGTAAAAGAACTAGTAGCTTACATATCAACTAGTGGTGAAAAAGATGCTGGAACA   900

Strain 13 GATGACTACATGTATTTTGGAATCAAAACAAAGGATGGAAAAACTCAAGAATGGGAAATGGACAACCCAGGAAATGATTTTATGACTGGAAGTAAAGACA  1000
Type Ve   GATGACTACATGTATTTTGGAATCAAAACAAAGGATGGAAAAACTCAAGAATGGGAAATGGACAACCCAGGAAATGATTTTATGACTGGAAGTAAAGACA  1000
Type N    GATGACTACATGTATTTTGGAATCAAAACAAAGGATGGAAAAACTCAAGAATGGGAAATGGACAACCCAGGAAATGATTTTATGACTGGAAGTAAAGACA  1000

Strain 13 CTTATACTTTCAAATTAAAAGATGAAAATCTAAAAATTGATGATATACAAAATATGTGGATTAGAAAAAGAAAATATACAGCATTCCCAGATGCTTATAA  1100
Type Ve   CTTATACTTTCAAATTAAAAGATGAAAATCTAAAAATTGATGATATACAAAATATGTGGATTAGAAAAAGAAAATATACAGCATTCCCAGATGTTTATAA  1100
Type N    CTTATACTTTCAAATTAAAAGATGAAAATCTAAAAATTGATGATATACAAAATATGTGGATTAGAAAAAGAAAATATACAGCATTCCCAGATGTTTATAA  1100

Strain 13 GCCAGAAAACATAAAGATAATAGCAAATGGAAAAGTTGTAGTAGACAAAGATATAAATGAGTGGATTTCAGGAAATTCAACTTATAATATAAAATAA     1197
Type Ve   GCCAGAAAACATAAAGGTAATAGCAAATGGAAAAGTTGTAGTGGACAAAGATATAAATGAGTGGATTTCAGGAAATTCAACTTATAATATAAAATAA     1197
Type N    GCCAGAAAACATAAAGGTAATAGCAAATGGAAAAGTTGTAGTGGACAAAGATATAAATGAGTGGATTTCAGGAAATTCAACTTATAATATAAAATAA     1197
```

Fig. S1 Alignment of *plc* sequence of the isolate with alpha toxin classified as alpha-toxin sequence type N. Nucleotides different from those of strain 13 and type Ve were shown as highlights and red, respectively. Sequences of strain 13 and type Ve (CP228) were obtained from GenBank with accession numbers BA000016 (1) and MH900561 (2), respectively.

```
Strain 13    MKRKICKALICATLATSLWAGASTKVYAWDGKIDGTGTHAMIVTQGVSIL    50
Type Ve      MKRKICKALICAALATSLWAGASTKVYAWDGKIDGTGTHAMIVTQGVSIL    50
Type N       MKRKICKALICAALATSLWAGASTKVYAWDGKIDGTGTHAMIVTQGVSIL    50

Strain 13    ENDLSKNEPESVRKNLEILKENMHELQLGSTYPDYDKNAYDLYQDHFWDP   100
Type Ve      ENDLSKNEPESVRKNLEILKENMHELQLGSTYPDYDKNAYDLYQDHFWDP   100
Type N       ENDLSKNEPESVRKNLEILKENMHELQLGSTYPDYDKNAYDLYQDHF*DP   100

Strain 13    DTDNNFSKDNSWYLAYSIPDTGESQIRKFSALARYEWQRGNYKQATFYLG   150
Type Ve      DTDNNFSKDNSWYLAYSIPDTGESQIRKFSALARYEWQRGNYKQATFYLG   150
Type N       DTDNNFSKDNSWYLAYSIPDTGESQIRKFSALARYEWQRGNYKQATFYLG   150

Strain 13    EAMHYFGDIDTPYHPANVTAVDSAGHVKFETFAEERKEQYKINTAGCKTN   200
Type Ve      EAMHYFGDIDTPYHPANVTAVDSAGHVKFETFAEERKEQYKINTAGCKTN   200
Type N       EAMHYFGDIDTPYHPANVTAVDSAGHVKFETFAEERKEQYKINTAGCKTN   200

Strain 13    EDFYADILKNKDFNAWSKEYARGFAKTGKSIYYSHASMSHSWDDWDYAAK   250
Type Ve      EAFYTDILKNKDFNAWSKEYARGFAKTGKSIYYSHASMSHSWDDWDYAAK   250
Type N       EAFYTDILKNKDFNAWSKEYARGFAKTGKSIYYSHASMSHSWDDWDYAAK   250

Strain 13    VTLANSQKGTAGYIYRFLHDVSEGNDPSVGKNVKELVAYISTSGEKDAGT   300
Type Ve      VTLANSQKGTAGYIYRFLHDVSEGNDPSVGKNVKELVAYISTSGEKDAGT   300
Type N       VTLANSQKGTAGYIYRFLHDVSEGNDPSVGKNVKELVAYISTSGEKDAGT   300

Strain 13    DDYMYFGIKTKDGKTQEWEMDNPGNDFMTGSKDTYTFKLKDENLKIDDIQ   350
Type Ve      DDYMYFGIKTKDGKTQEWEMDNPGNDFMTGSKDTYTFKLKDENLKIDDIQ   350
Type N       DDYMYFGIKTKDGKTQEWEMDNPGNDFMTGSKDTYTFKLKDENLKIDDIQ   350

Strain 13    NMWIRKRKYTAFPDAYKPENIKIIANGKVVVDKDINEWISGNSTYNIK     398
Type Ve      NMWIRKRKYTAFPDVYKPENIKVIANGKVVVDKDINEWISGNSTYNIK     398
Type N       NMWIRKRKYTAFPDVYKPENIKVIANGKVVVDKDINEWISGNSTYNIK     398
```

Fig. S2 Alignment of deduced amino acid sequence of the isolate with alpha toxin classified as alpha-toxin sequence type N. Amino acids different from those of strain 13 and type Ve were shown as highlights and red, respectively, and deficient amino acids were shown as gray and underlined with broken lines. An asterisk shows stop codon. Amino acid sequences of strain 13 and type Ve (CP228) were obtained from GenBank with accession numbers BA000016 (1) and MH900561 (2), respectively.

Table S1 Number of PLC-negative *C. perfringens* type F isolates from influents and effluents of WWTP-A and WWTP-B.

		Influent		Effluent	
		WWTP-A	WWTP-B	WWTP-A	WWTP-B
2016	7	0	0	–	–
	8	2	0	–	–
	9	0	0	–	–
	10	0	0	–	–
	11	0	0	–	–
	12	0	0	–	–
2017	1	0	0	–	–
	2	0	0	–	–
	3	0	0	–	–
	4	1	1	–	–
	5	1	0	–	–
	6	1	1	–	–
	7	0	0	–	–
	8	0	0	–	–
	9	0	1	–	–
	10	0	0	–	–
	11	0	0	–	–
	12	2	0	–	–
2018	1	0	1	–	–
	2	0	1	–	–
	3	1	0	–	–
	4	2	2	–	–
	5	0	1	–	–
	6	2	0	–	–
	7	1	0	–	–
	8	1	0	0	4
	9	0	0	0	0
	10	1	0	3	1
	11	2	0	0	1
	12	3	0	0	0
2019	1	1	0	1	0
	2	1	1	0	0
	3	2	0	0	0
	4	0	1	0	1
	5	0	1	0	0
	6	0	1	0	1
	7	0	0	0	0
	8	0	1	0	0
	9	0	1	0	0
	10	0	1	0	0
	11	1	1	0	0
	12	0	0	0	1
2020	1	2	0	0	0
Total		27	16	4	9

−: not tested.

Reference

1. Shimizu T, Ohtani K, Hirakawa H, Ohshima K, Yamashita A, Shiba T, Ogasawara N, Hattori M, Kuhara S, Hayashi H. 2002. Complete genome sequence of *Clostridium perfringens*, an anaerobic flesh-eater. Proc Natl Acad Sci U S A 99:996–1001. https://doi: 10.1073/pnas.022493799.
2. Matsuda A, Aung MS, Urushibara N, Kawaguchiya M, Sumi A, Nakamura M, Horino Y, Ito M, Habadera S, Kobayashi N. 2019. Prevalence and genetic diversity of toxin genes in clinical isolates of *Clostridium perfringens*: coexistence of alpha-toxin variant and binary enterotoxin genes (*bec*/*cpile*). Toxins 11:326. https://doi: 10.3390/toxins11060326.
